# On the Interaction Between SMARCAL1 and BRG1

**DOI:** 10.3389/fcell.2022.870815

**Published:** 2022-06-16

**Authors:** Deepa Bisht, Ketki Patne, Radhakrishnan Rakesh, Rohini Muthuswami

**Affiliations:** Chromatin Remodeling Laboratory, School of Life Sciences, JNU, New Delhi, India

**Keywords:** SMARCAL1-BRG1 interaction, SMARCAL1, BRG1, protein-protein interaction, SIOD, CSS4

## Abstract

SMARCAL1 and BRG1, both classified as ATP-dependent chromatin remodeling proteins, play a role in double-strand break DNA damage response pathways. Mutations in SMARCAL1 cause Schimke Immuno-osseous Dysplasia (SIOD) while mutations in BRG1 are associated with Coffin-Siris Syndrome (CSS4). In HeLa cells, SMARCAL1 and BRG1 co-regulate the expression of *ATM*, *ATR,* and RNAi genes on doxorubicin-induced DNA damage. Both the proteins are found to be simultaneously present on the promoter of these genes. Based on these results we hypothesized that SMARCAL1 and BRG1 interact with each other forming a complex. In this paper, we validate our hypothesis and show that SMARCAL1 and BRG1 do indeed interact with each other both in the absence and presence of doxorubicin. The formation of these complexes is dependent on the ATPase activity of both SMARCAL1 and BRG1. Using deletion constructs, we show that the HARP domains of SMARCAL1 mediate interaction with BRG1 while multiple domains of BRG1 are probably important for binding to SMARCAL1. We also show that SIOD-associated mutants fail to form a complex with BRG1. Similarly, CSS4-associated mutants of BRG1 fail to interact with SMARCAL1, thus, possibly contributing to the failure of the DNA damage response pathway and pathophysiology associated with SIOD and CSS4.

## Introduction

The ATP-dependent chromatin remodeling proteins use the energy released from ATP hydrolysis to remodel nucleosomes, a process necessary for gene regulation as well as DNA damage repair (Osley et al., 2007; Clapier and Cairns, 2009; Hargreaves and Crabtree, 2011). The ATP-dependent chromatin remodeling proteins are grouped into helicases family due to the presence of seven helicase motifs that confer the DNA binding and ATP hydrolysis properties (Flaus et al., 2006). However, none of these proteins possess any helicase activity (Côté et al., 1994; Muthuswami et al., 2000). Instead, they use the energy liberated from ATP hydrolysis in altering the position of the nucleosome and maintaining chromatin architecture (Clapier and Cairns, 2009). The only exception is the INO80 complexes that show helicase activity due to the presence of Rvb1 and Rvb2 proteins (Shen et al., 2000; Conaway and Conaway, 2009). Phylogenetic analysis has identified six sub-families of which BRG1 is placed in the Snf2 class and SMARCAL1 has been classified as a distant member of the ATP-dependent chromatin remodeling protein family (Flaus et al., 2006).

BRG1 is a transcriptional modulator forming many complexes within the cell (Trotter and Archer, 2008). The protein also plays a role in DNA double-strand break repair where it is recruited to the break site via the interaction between bromodomain of BRG1 and acetylated H3 (Park et al., 2006; Lee et al., 2010; Kwon et al., 2015). This interaction is essential for remodeling nucleosomes at the site of DNA damage and for spreading the acetylated H3 (Lee et al., 2010). BRG1 has been also shown to co-localize with γH2AX (Lee et al., 2010). Mutations in BRG1 are associated with lung, liver, prostate, breast, and pancreatic cancers (Wong et al., 2000; Reisman et al., 2003; Roy et al., 2015; Wu et al., 2017; Muthuswami et al., 2019; Wang et al., 2020). In addition, mutations in BRG1 also leads to Coffin-Siris Syndrome (CSS4), an autosomal dominant disorder, that is characterized by kidney abnormalities and azoospermia (Tsurusaki et al., 2012).

SMARCAL1 is an annealing helicase that promotes replication fork regression when double-strand breaks are induced in DNA (Bansbach et al., 2009; Postow et al., 2009; Bétous et al., 2012). During DNA damage the protein is recruited to the replication fork by RPA and the ATPase activity is used for re-annealing the single-stranded DNA. This protein too co-localizes with γH2AX (Bansbach et al., 2009; Ciccia et al., 2009). Mutations in SMARCAL1 cause Schimke Immuno-osseous Dysplasia (SIOD), an autosomal recessive disorder characterized by renal dysfunction, spondyloepiphyseal dysplasia, and T-cell immunodeficiency (Boerkoel et al., 2002).

Previously, we have shown that SMARCAL1 and BRG1 are co-regulated such that downregulation of SMARCAL1 results in reduced expression of BRG1 and downregulation of BRG1 results in repression of SMARCAL1 expression (Haokip et al., 2016). We further showed that this co-regulation is important for the functioning of the DNA damage response pathway as SMARCAL1 and BRG1 transcriptionally co-regulate the expression of *ATM* and *ATR* in HeLa cells (Sethy et al., 2018). They also co-regulate the expression of *DROSHA*, *DGCR8,* and *DICER*, thus, transcriptionally regulating the expression of damage response ncRNA that mediate the formation of 53BP1 foci (Patne et al., 2017).

The experimental evidence that both SMARCAL1 and BRG1 are present together on gene promoters led us to hypothesize that these proteins interact with each other forming a complex. In this paper, we present evidence that SMARCAL1 and BRG1 interact with each other both in the absence and presence of doxorubicin-induced DNA damage. We show that this interaction is dependent on the ATPase activity of both the ATP-dependent chromatin remodeling proteins. This interaction is abrogated in SIOD-associated and CSS4-associated mutants suggesting that the pleiotropic effects observed in SIOD and CSS4 patients could also stem from the impaired complex formation by SMARCAL1 and BRG1.

## Material and Methods

**Chemicals:** All chemicals and reagents required for cell culture were purchased from Hi-media (United States). Sodium bicarbonate, Hoechst 33342, doxorubicin was purchased from Sigma-Aldrich (United States). Cell culture-grade dishes were purchased from Corning (Germany). For western blotting, PVDF membrane was purchased from Merck-Millipore (United States). X-ray sheets, fixer, and developer were purchased from Kodak (United States). Luminol, Coumaric acid, and hydrogen peroxide were purchased from Hi-media (United States). Turbofect was purchased from Thermo Scientific (United States). Protein G Beads was purchased from Merck-Millipore (United States).

**Antibodies:** Antibodies against BRG1 (Catalog #B8184) and γH2AX (Catalog #H5912) were purchased from Sigma-Aldrich (United States). SMARCAL1 antibody was custom raised against purified recombinant HARP1 domain (Catalog # 106014; Merck, India) (Haokip et al., 2016). TRITC and FITC-conjugated anti-mouse and anti-rabbit (Catalog# RTC2 and FTC3), as well as HRP-conjugated anti-rabbit IgG (Catalog#HPO3) and anti-mouse IgG (Catalog# HPO5) antibodies, were purchased from Merck (India).

**Cell culture:** HeLa and THP-1 cells, purchased from NCCS, Pune, were maintained in DMEM and RPMI media, respectively, containing 10% (v/v)) FBS and an antibiotic cocktail of penicillin, streptomycin, and amphotericin.

**Co-immunoprecipitation:** HeLa cells were grown to 70–80% confluency and resuspended in 300 μL of RIPA buffer (250 mM NaCl, 50 mM Tris. Cl pH 8.0, 2 mM EDTA, 1 mM PMSF, 1X protease inhibitor, 1% NP-40). After incubating at 4°C for 15 min, the cells were lysed by sonication (20 s ON; 30 s OFF- 10 cycles). The supernatant was obtained by centrifugation at 12,000 rpm for 30 min at 4°C. The process was repeated with the cell pellet and the combined supernatant was stored at −80°C till required.

The prepared extract was incubated with 20 μL protein G beads for 1 h at 4°C. After incubation, the supernatant was collected by centrifugation at 2000 rpm and quantified using Bradford reagent. 2 μg of antibodies was added to ∼300 μg pre-cleared extract and incubated using a rotator at 4°C for 16 h. The next day, 20 μL protein G beads blocked with salmon sperm ssDNA and BSA was added to the extract-antibody mix and incubated for 4 h. The beads were then centrifuged at 2000 rpm and washed 4 times with lysis buffer. For analysis, the beads were boiled in Laemmli buffer (2% (v/v) SDS, 10% (v/v) glycerol, 60 mM Tris. Cl, pH 6.8) for 15 min. The supernatant was loaded on either 6 or 7% SDS polyacrylamide gel with a pre-stained loading marker and processed for western blotting.

**Western Blotting:** The gel was transferred on to PVDF membrane. After transfer, the membrane was washed 1X PBS buffer and blocked using 5% (w/v) BSA in 1X PBST (PBS containing 0.05% (v/v) Tween 20) for 1 h at 37°C. After blocking, the membrane was incubated with primary antibody solution with recommended dilution at 4°C overnight. The next day, the membrane was washed 4 times in 1X PBST for 5 min each on a rocker. The membrane was next incubated with secondary antibody solution (1:4000 dilution) for 1 h at 37°C. After incubation, the membrane was washed 4 times in 1X PBST for 5 min each on a rocker. A final wash was given with 1X PBS, and the membrane was developed using an Enhanced Chemiluminescence solution.

**Oligonucleotides:** The primers for cloning were designed from Ensemble Database and NCBI nucleotide and were synthesized from GCC Biotech (India). The primer sequences are provided in Supplementary Table S1.

**Constructs:** pcDNA3.1 Zeo-LAP-SMARCAL1 vector was cloned as explained in Haokip et al. (Haokip et al., 2016). BRG1 was sub-cloned from BJ5-BRG1 into pcDNA3.1 Zeo-LAP. Deletion constructs of SMARCAL1 and BRG1 were made using primers (Supplementary Table S1) spanning the deletion sites and amplified by PCR using Pfu DNA polymerase.

SMARCAL1 mutants corresponding to those observed in SIOD patients and BRG1 mutants corresponding to CSS4 patients were cloned as explained previously (Gupta et al., 2015; Sethy et al., 2018).

**Transfections:** HeLa cells were seeded in a 35 mm cell culture grade dish with a glass coverslip and incubated for 12 h so that they reached 60% confluency. THP-1 cells (10^6^ cells/ml) were seeded 35 mm cell culture grade dish with a glass coverslip and differentiated using PMA. For each 35 mm dish, 1.5 μg plasmid DNA was mixed with 3 μL Turbofect reagent and transfection was done as per the manufacturer’s instructions.

**Immunofluorescence:** For immunofluorescence, the cells were seeded on a coverslip placed inside the 35 mm dish. The cells were washed twice with 1X PBS and fixed with ice-cold acetone and methanol (1:1) for 15 min. The acetone: methanol solution was discarded and replaced with ice-cold 1X PBS added gently. After a brief incubation on ice, Triton X-100 (SRL India) at final concentration of 0.5% (v/v) was added for permeabilization. The cells were incubated in dark at 4°C for 10 min. After permeabilization, the coverslip was blocked using 2% (w/v) BSA for 1 h at 37°C. Subsequently, the coverslips were incubated with primary antibody solution (1:250) overnight at 4°C. The next day, the coverslip was washed 4–5 times in 1X PBS containing 0.2% Triton X-100 for 5 min each. After washing, the coverslip was incubated in secondary antibody (1:1000 dilution) solution and Hoechst 33,342 dye for 30–45 min at 37°C. The coverslips were washed 4–5 times with 1X PBS containing Triton X-100 for 5 min each and studied under a microscope.

**Fixed cell FRET:** These experiments were performed using Nikon A1R HD confocal microscope equipped with all four lasers (405, 488, 567, and 637 nm). The pictures were taken with a 60X objective lens with 1.5 times zoom. The pinhole was set at one Airy unit. The laser power was kept 100% for bleaching and 5% for capturing the image. The ROI was annotated, and a pre-bleached image was captured for 10 s using 488 and 561 nm lasers. The same area was bleached for the 30 s using 100% power of 561 nm laser. The post-bleached image was also captured using both the lasers used for pre-bleached images. The change in the pre-bleached and post-bleached donor intensities was measured using Nikon A1R analysis software. The FRET efficiencies were calculated using the formula (FRET efficiency = (1- Donor pre/Donor post) and plotted with the help of Sigma plot version 10.0.

**SMARCAL1 and BRG1 co-immunoprecipitate both in the absence and presence of DNA damage:** The ChIP-sequencing were performed using HeLa cells. The raw reads of ChIP-sequencing data (GSE137250) were processed on the Galaxy (https://usegalaxy.org) platform. The adaptor sequences were trimmed from raw reads using trimmomatic (version 0.36.5), followed by quality control analysis using FastQC. The processed reads were aligned to the reference genome (hg38) with the BOWTIE2 tool with default settings. The aligned files were marked for duplicates by PicardMarkduplicates and filtered on bit-wise flags by SAM tools on the Galaxy platform. Only paired-end reads that were mapped in proper pair were selected for peak calling. Biological replicates of SMARCAL1 and BRG1 were merged in a single BAM file before peak calling. Peak calling was performed by MACS2 (Version 2.1.1.20160309.0) with default settings. Gene annotation and gene ontology was done using HOMER and clusterProfiler respectively. Venn diagrams were plotted using software (http://bioinformatics.psb.ugent.be/software).

The motif-based sequence analysis was performed with the FASTA format of the identified SMARCAL1 peaks using the MEME Suite tool (Bailey et al., 2009). The motif with the lowest E-value was selected for further study.

**Statistical analysis:** Statistical analyses were performed by SigmaPlot version 10. Pearson’s correlation test was used to compare the distribution of data in studies.

## Results

**FIGURE 1:**
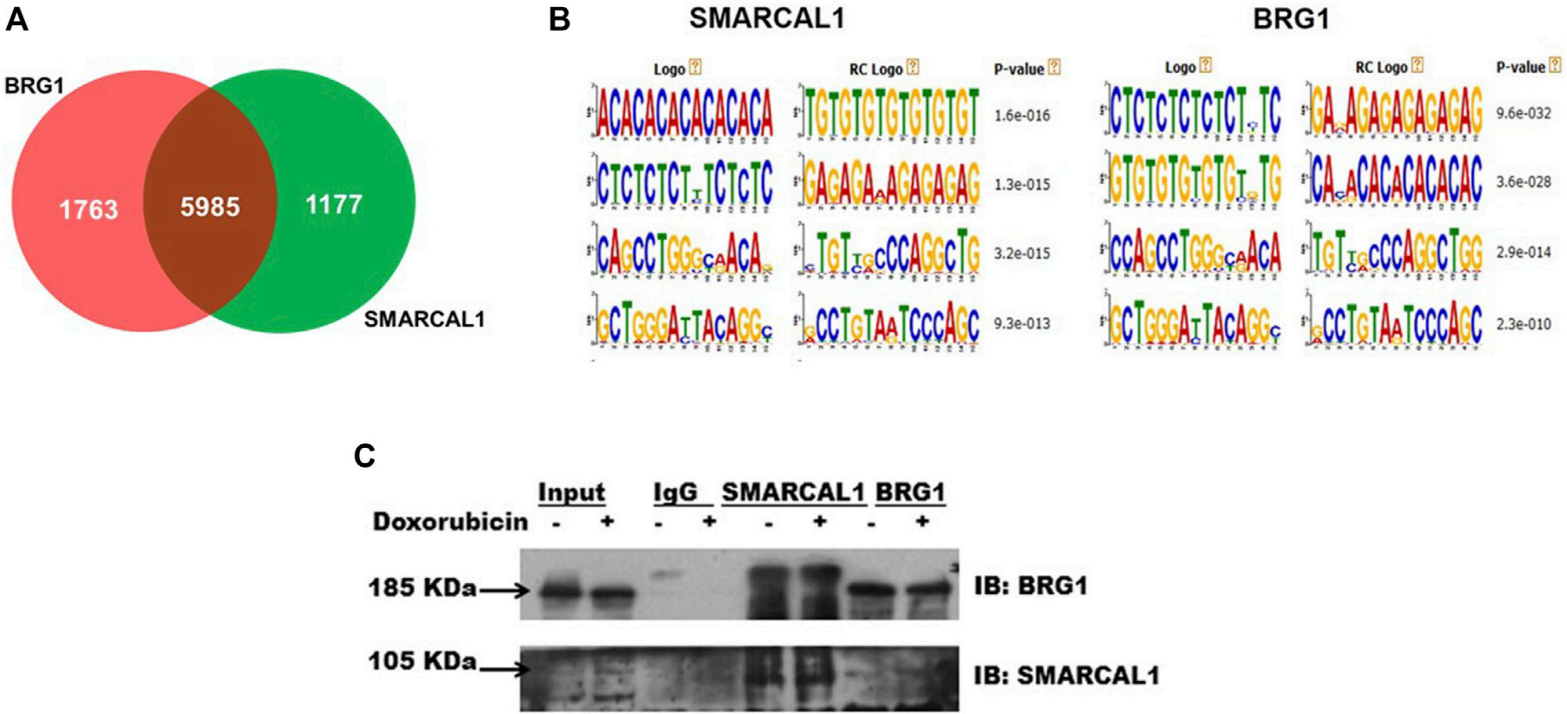
SMARCAL1 and BRG1 co-immunoprecipitate, in the absence and presence of DNA damage: Genome-wide occupancy of SMARCAL1 and BRG1 identified by ChIP-seq analysis **(A)**. Venn diagram showing the intersection of the majority of genes **(B)**. Identical DNA motifs occupied by SMARCAL1 and BRG1 **(C)** Co-immunoprecipitation experiments were performed with anti SMARCAL1 and anti-BRG1 in untreated and treated protein samples with 2 μM doxorubicin for 10 min. The pull-down protein samples were probed for both proteins. IgG antibody was used for negative control. HeLa cells were used for the experiments. The experiment was performed using two independent biological samples and a representative blot has been included.

**SMARCAL1 and BRG1 co-immunoprecipitate, in the absence and presence of DNA damage:** Analysis of the ChIP-seq data performed using HeLa cells (GSE137250) showed that SMARCAL1 was present on 7161 genes while BRG1 was present on 7747 genes. When all the genes occupied by both these proteins were compared and intersected, a set of 6000 genes were identified where both SMARCAL1 and BRG1 were found to be present (Figure 1A). Further, motif analysis using MEME-Suite showed SMARCAL1 and BRG1 to be present on either identical or similar DNA motifs across the various genomic locations. (Figure 1B). Experimentally, we had previously shown that SMARCAL1 and BRG1 are present simultaneously on *ATM*, *ATR*, *DROSHA*, *DGCR8*, and *DICER* promoters (Patne et al., 2017; Sethy et al., 2018). Based on these data, we hypothesized that SMARCAL1 and BRG1 interact with each other.

To test the hypothesis, we performed co-immunoprecipitation experiments in the absence and presence of doxorubicin using HeLa extracts. The study was performed with HeLa cells both in the absence and presence of doxorubicin. Doxorubicin intercalates between bases of DNA and impedes topoisomerase II movement. This results in double-strand break which is repaired by the double-strand break repair pathway (Yang et al., 2014; Yang et al., 2015).

Co-immunoprecipitation experiments showed that SMARCAL1 and BRG1 interact both in the absence and presence of doxorubicin-induced DNA damage (Figure 1C). Further, the interaction between SMARCAL1 and BRG1 was observed both in the absence and presence of apoptosis.

**SMARCAL1 and BRG1 interact with each other both in the absence and presence of DNA damage:** The co-immunoprecipitation experiments do not provide information on whether these proteins are interacting directly or indirectly. Therefore, co-localization and FRET were used to delineate the interaction between BRG1-SMARCAL1 in the absence and presence of 2 μM doxorubicin. As the antibodies for SMARCAL1 and BRG1 were not compatible to screen for endogenous interaction, GFP-SMARCAL1 was overexpressed in HeLa cells and the interaction of the overexpressed protein with endogenous BRG1 was monitored. 48 h post-transfection, the cells were treated with 2 μM doxorubicin for 10 min.

As both SMARCAL1 and BRG1 have been shown to co-localize with γH2AX at the site of DNA damage, we have used this co-localization as a positive control for our studies (Park et al., 2006; Postow et al., 2009). Therefore, we first probed SMARCAL1-γH2AX and BRG1-γH2AX interactions by transfecting HeLa cells with constructs expressing either GFP-BRG1 or GFP-SMARCAL1. In untreated cells, γH2AX foci could not be detected whereas in 2 μM doxorubicin-treated cells γH2AX foci co-localized with both SMARCAL1 and BRG1. In contrast, no interaction was observed in cells transfected with empty GFP-vector in untreated as well as treated conditions (Supplementary Figure 1A-D).

**FIGURE 2:**
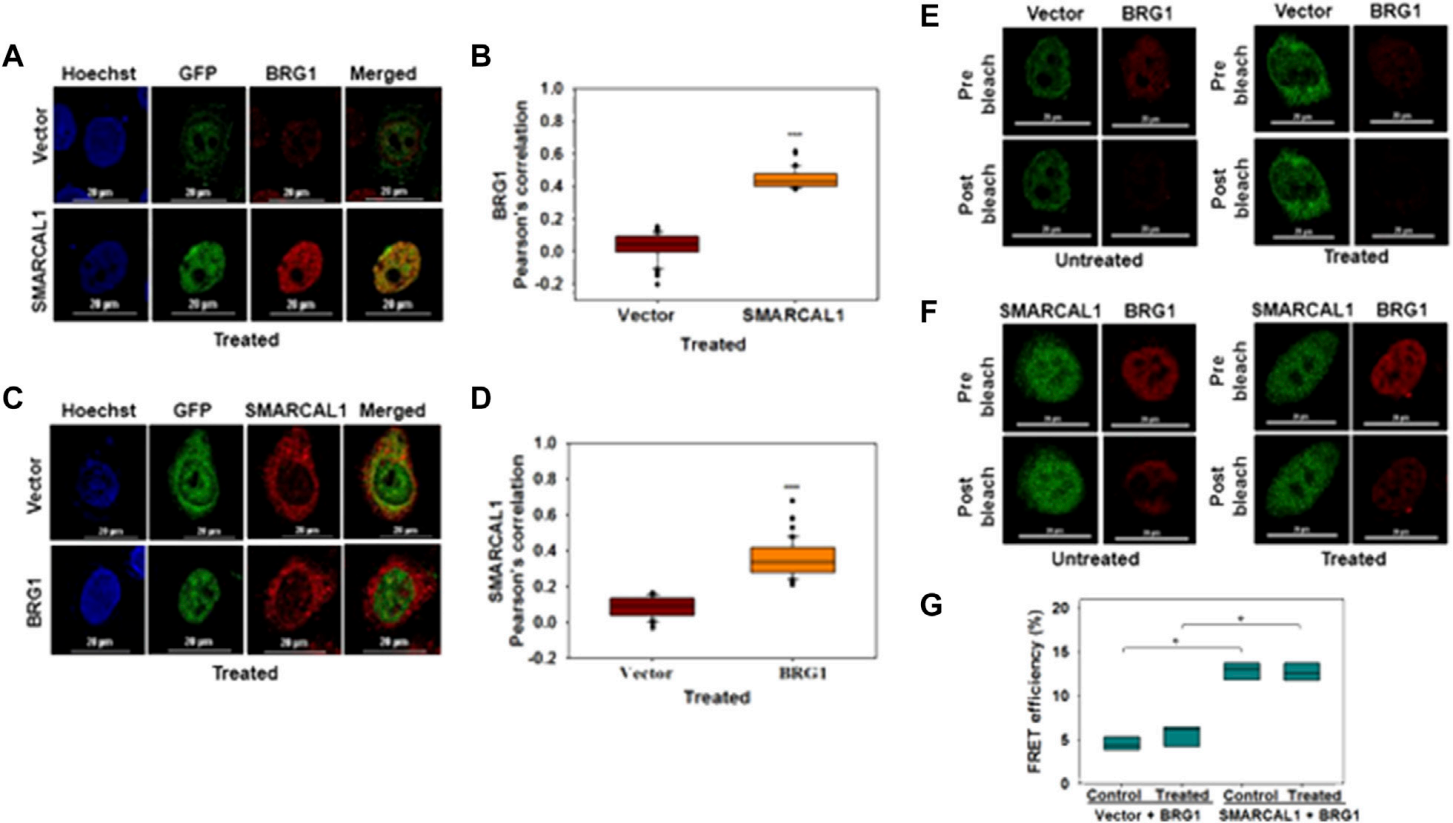
SMARCAL1 and BRG1 interact with each other both in the absence and presence of DNA damage **(A)**. Co-localization between GFP-SMARCAL1 and endogenous BRG1 was monitored in the presence of doxorubicin-induced DNA damage in HeLa cells **(B)**. Pearson’s coefficient for the co-localization of GFP SMARCAL1 with BRG1 **(C)**. Co-localization between GFP-BRG1 and endogenous SMARCAL1 was monitored in the presence of doxorubicin-induced DNA damage in HeLa cells **(D)**. Pearson’s coefficient the interaction of GFP-BRG1 with SMARCAL1 **(E)**. Acceptor Photobleach FRET of GFP-vector alone signal after bleaching endogenous BRG1 in the absence and presence of doxorubicin treatment **(F)**. Acceptor photobleach FRET of GFP-SMARCAL1 signal after bleaching endogenous BRG1 in the absence and presence of doxorubicin treatment **(G)**. Quantitation of the FRET efficiency for the interaction of GFP-SMARCAL1 with BRG1. In all the co-localization experiments, HeLa cells were treated with 2 μM doxorubicin for 10 min and n ≥ 90 cells for GFP-SMARCAL1 and BRG1 and n ≥ 40 cells for GFP-BRG1 and SMARCAL1 were analyzed. In the FRET experiments, n ≥ 8 cells were analyzed and 2 μM doxorubicin treatment was given for 10 min. In the FRET experiments, n ≥ 8 cells were analyzed and 2 μM doxorubicin treatment was given for 10 min. Star indicates significance with *p-value < 0.05, **p-value < 0.005, ***p value <0.0001. The scale in the images is 20 μm.

Next, we probed the co-localization of SMARCAL1 and BRG1. HeLa cells were transfected with GFP-SMARCAL1, and co-localization studies showed that SMARCAL1 and BRG1 were present in proximity within the cell both in the absence and presence of DNA damage (Supplementary Figure 2A and Figure 2A, respectively). The Pearson’s coefficient in the absence of DNA damage was 0.49 ± 0.08 while it was 0.46 ± 0.07 in the presence of DNA damage (Supplementary Figure 2B and Figure 2B, respectively), indicating that there is no change in co-localization as a function of DNA damage. Co-localization studies were performed in THP-1 cells also after differentiation using PMA. In this case, we found that BRG1 and SMARCAL1 co-localization was more prominent in the presence of DNA damage (Supplementary Figure 3A-D).

In the reverse experiment, HeLa cells were transfected with GFP-BRG1 and the interaction of the overexpressed protein with endogenous SMARCAL1 was studied. These experiments also showed that SMARCAL1 and BRG1 co-localize in the same space within the nucleus both in the absence and presence of doxorubicin-induced DNA damage (Supplementary Figure 2C and Figure 2C respectively). The Pearson’s coefficient in the absence of DNA damage was 0.38 ± 0.12 while it was 0.37 ± 0.1 in the presence of DNA damage, once again indicating that the interaction did not alter as a function of DNA damage (Supplementary Figure 2D and Figure 2D respectively). A similar result was also obtained with THP-1 cells (Supplementary Figure 3E-H).

To further confirm the interaction we used acceptor photobleaching FRET (Shimi et al., 2004; Weems et al., 2015). The FRET experiment showed that GFP-SMARCAL1 and BRG1 interact with each other with a FRET efficiency of 12.84 ± 1.03% in the absence of DNA damage showing a statistically significant increase of approximately 3-fold as compared to the vector control (Figures 2E,F). The FRET efficiency in the presence of DNA damage the efficiency was 13.3 ± 2.72%, which was approximately 2-fold higher as compared to the vector control (Figures 2E,F). The FRET efficiency was also calculated in THP-1 cells also showed that GFP-SMARCAL1 and BRG1 interact with each other with a FRET efficiency of 68.2 ± 11.94% in control cells and 73.14 ± 13.23% in the presence of DNA damage (Supplementary Figure 4A-C). These FRET efficiencies were found to be statistically significant over the vector alone controls.

In the reverse experiment, BRG1 was overexpressed, and FRET with endogenous SMARCAL1 was monitored. Here, too, the FRET efficiency was 12.56 ± 1.04% in the absence of DNA damage and was 13.11 ± 2.16% in doxorubicin-treated cells (Supplementary Figure 2E,F). These FRET efficiencies were once again found to be statistically significant as compared to the vector control (Supplementary Figure 2E,F).

Thus, both the co-localization and FRET results suggest that SMARCAL1 and BRG1 physically interact with each other. This data has been used in all the further analyses.

**The ATPase activity of SMARCAL1 and BRG1 is required for their co-localization:** As both BRG1 and SMARCAL1 are ATP-dependent chromatin remodelers, the importance of their ATPase activity in mediating the interaction with each other was next investigated. To understand the importance of ATPase activity for the interaction, K464A mutant of SMARCAL1 and K785R mutant of BRG1 transfected into HeLa cells as these K464A in SMARCAL1 and K785R in BRG1 is required for ATPase activity of these proteins (Khavari et al., 1993; Gupta et al., 2015).

**FIGURE 3:**
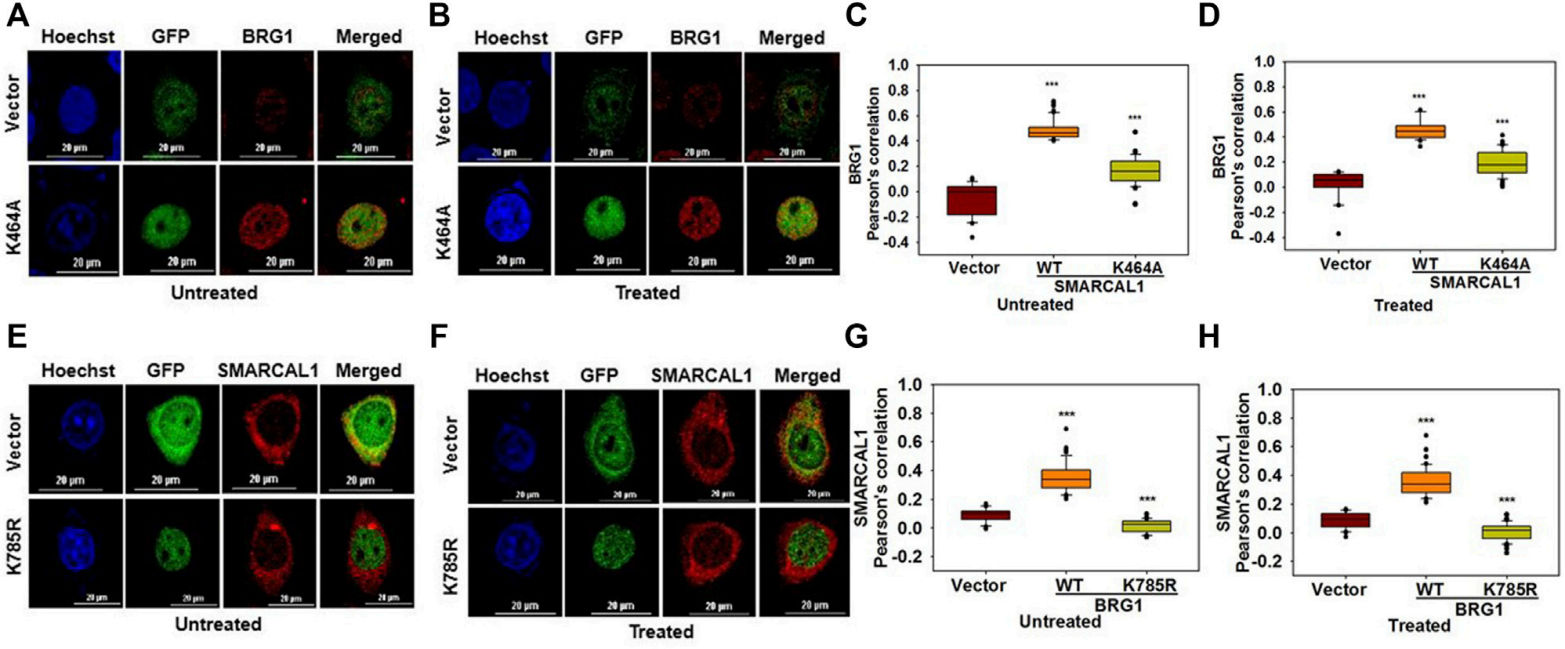
The ATPase activity of SMARCAL1 and BRG1 is required for their co-localization **(A)**. Co-localization of GFP-SMARCAL1 K464A with BRG1 in the absence of doxorubicin treatment **(B)**. Co-localization of GFP-SMARCAL1 K464A with BRG1 in the presence of 2 μM doxorubicin treatment for 10 min **(C)**. Pearson’s coefficient for the interaction in the absence of doxorubicin treatment **(D)**. Pearson’s coefficient for the interaction in the presence of doxorubicin treatment **(E)**. Co-localization of GFP-BRG1 K785R with SMARCAL1 in the absence of doxorubicin treatment **(F)**. Co-localization of GFP-BRG1 K785R with SMARCAL1 in the presence of 2 μM doxorubicin treatment for 10 min **(G)**. Pearson’s coefficient for the interaction in the absence of DNA damage **(H)** Pearson’s coefficient for the interaction in the presence of DNA damage. In all these experiments, HeLa cells were treated with 2 μM doxorubicin for 10 min and n ≥ 40 cells for GFP-SMARCAL1 K464A and BRG1 and n ≥ 60 cells for GFP-BRG1 K785R and SMARCAL1 were analyzed. Star indicates significance with *p-value < 0.05, **p-value < 0.005, ***p value <0.0001. The wild-type data used in the analysis has been shown in Figure 2 The scale in the images is 20 μm.

HeLa cells were transfected with GFP-SMARCAL1 K464A, as this mutant lacks ATPase activity (Gupta et al., 2015), and its interaction was studied with the endogenous wild-type BRG1. The co-localization showed a decrease in the interaction of SMARCAL1 K464A and BRG1 compared to the wild type SMARCAL1 and BRG1 both in the absence and presence of DNA damage (Figure 3A–D). Similarly, the co-localization between GFP-BRG1 K785R and endogenous wild-type SMARCAL1 also decreased significantly both in the absence and presence of DNA damage (Figure 3E–H).

The results show that the ATPase activity of both the proteins was needed for co-localization with each other.

**FIGURE 4:**
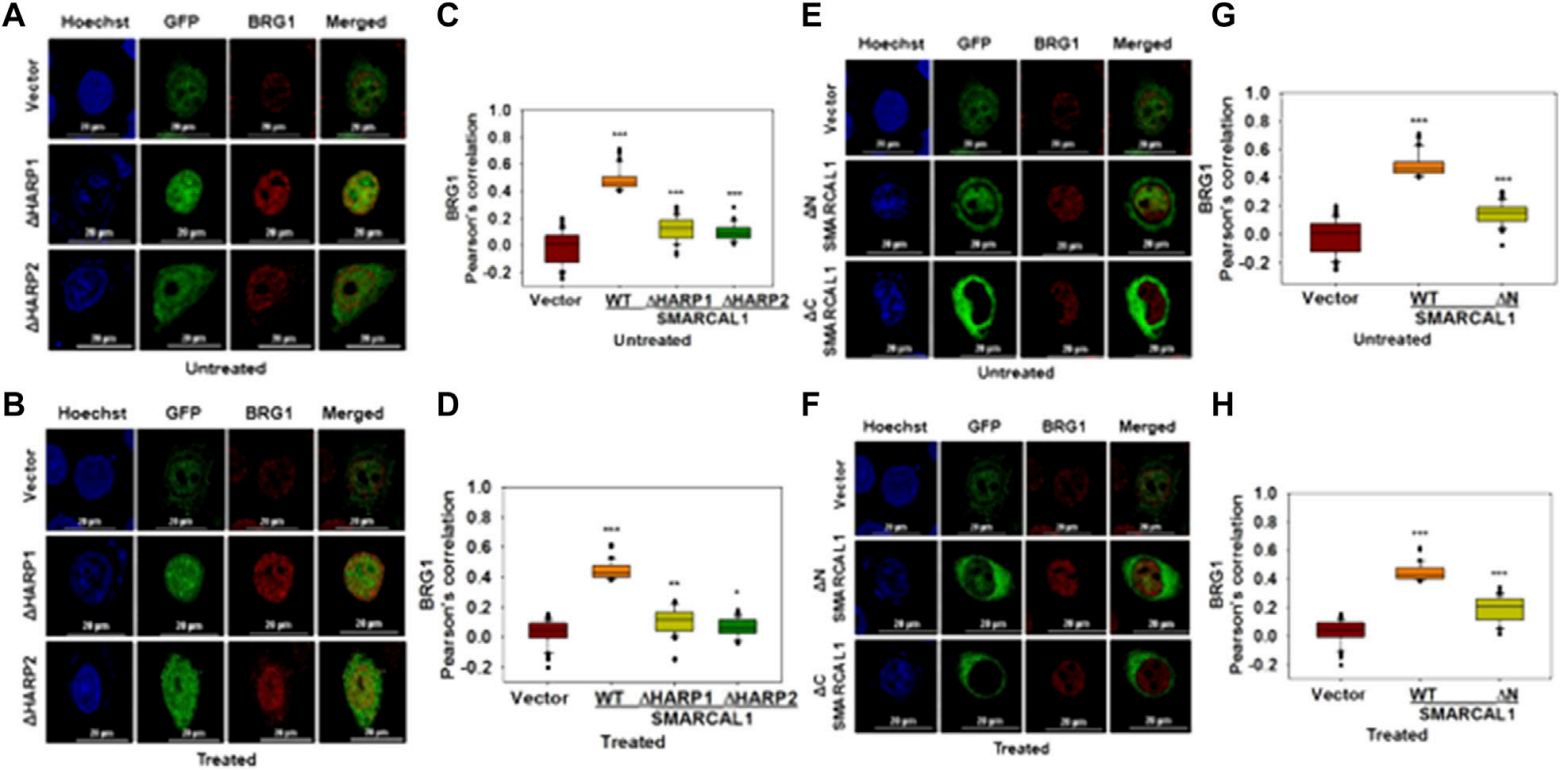
HARP domains of SMARCAL1 are required for interaction with BRG1 **(A)**. Co-localization of GFP-ΔHARP1 and GFP-ΔHARP2 with BRG1 in the absence of doxorubicin treatment **(B)**. Co-localization of GFP-ΔHARP1 and GFP-ΔHARP2 with BRG1 in the presence of 2 μM doxorubicin treatment for 10 min **(C)**. Pearson’s coefficient for the interaction of GFP-ΔHARP1 and GFP-ΔHARP2 with BRG1 in the absence of doxorubicin treatment **(D)**. Pearson’s coefficient for the interaction of GFP-ΔHARP1 and GFP-ΔHARP2 with BRG1 in the presence of 2 μM doxorubicin treatment for 10 min **(E)**. Co-localization of GFP-ΔN and GPF-ΔC with endogenous BRG1 in the absence of doxorubicin treatment **(F)**. Co-localization of GFP-ΔN and GPF-ΔC with endogenous BRG1 in the presence of 2 μM doxorubicin treatment for 10 min **(G)**. Pearson’s coefficient for the interaction of GFP-ΔN and GFP-ΔC with endogenous with BRG1 in the absence of doxorubicin treatment **(H)**. Pearson’s coefficient for the interaction of GFP-ΔN and GFP-ΔC with endogenous with BRG1 in the presence of doxorubicin treatment. In all these experiments, HeLa cells were treated with 2 μM doxorubicin for 10 min and n ≥ 40 cells were analyzed. Star indicates significance with *p-value < 0.05, **p-value < 0.005, ***p value <0.0001. The wild-type data used in the analysis has been shown in Figure 2. The scale in the images is 20 μm.

**The HARP domains of SMARCAL1 are required for interaction with BRG1:** To delineate the domains of SMARCAL1 required for interaction with BRG1, four deletion constructs were made - ΔHARP1 lacking the HARP1 domain, ΔHARP2 lacking the HARP2 domain, ΔN lacking the entire N-terminal domain, and ΔC lacking the C-terminal domain containing the helicase motifs (Supplementary Figure 5A). Each of these mutants was transfected into HeLa cells and the interaction with BRG1 in the absence and presence of doxorubicin-induced DNA damage was studied using co-localization. Of the four deletion constructs, ΔC localized only to the cytoplasm both in the absence and presence of DNA damage (Figure 4E,F). It has been reported that a nuclear localization signal is presented within the helicase motifs (Coleman et al., 2000). Therefore, ΔC possibly lacks the signal to move into the nucleus. The interaction of BRG1 with ΔHARP1, ΔHARP2, and ΔN was found to be impaired with the Pearson’s correlation maximally reduced in the case of ΔHARP2 deletion construct both in the absence and presence of DNA damage (Figure 4A–H), thus, indicating that the HARP domains might be playing an important role in SMARCAL1-BRG1 interaction.

**FIGURE 5:**
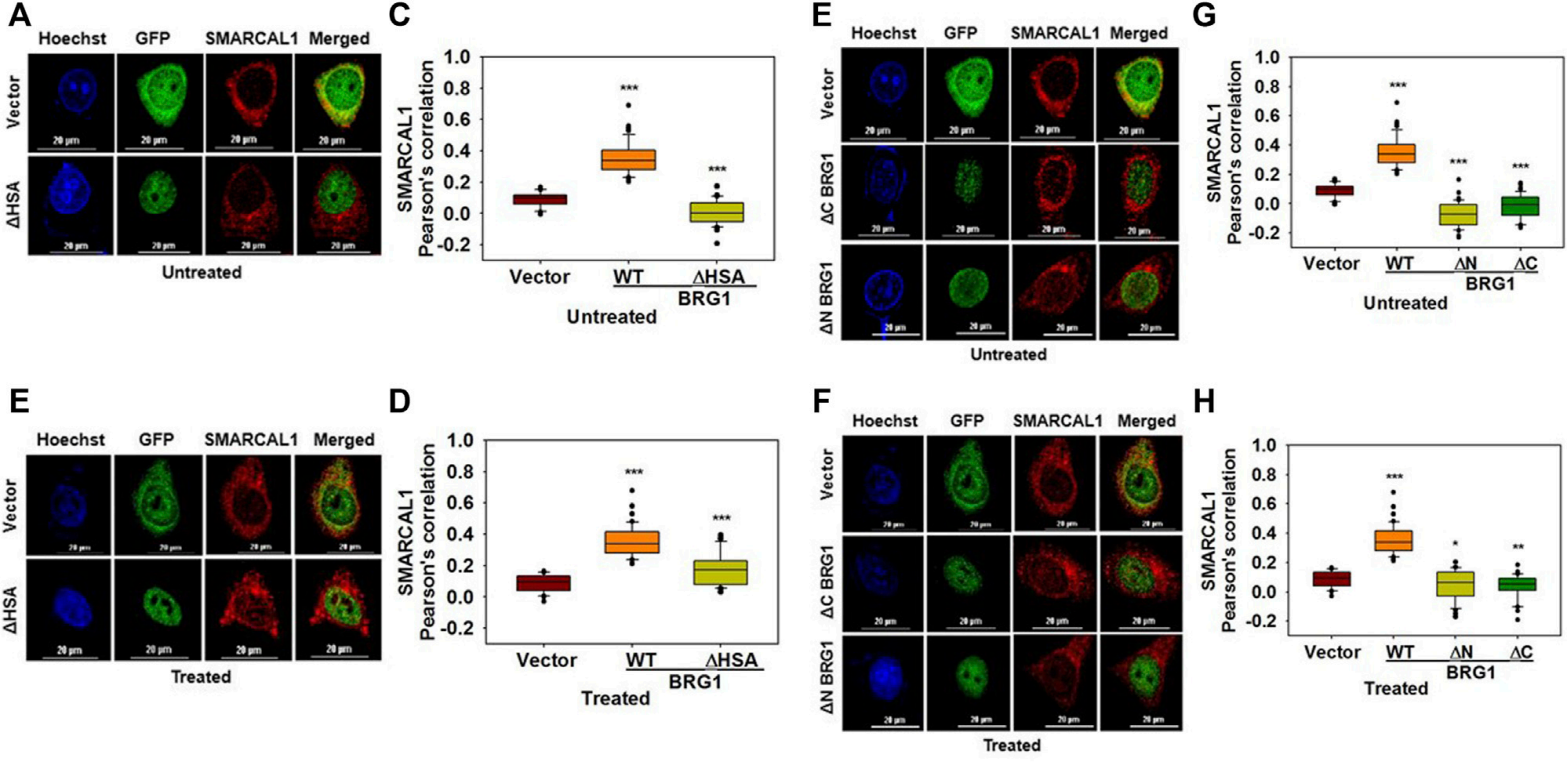
Multiple regions of BRG1 are required for interaction with SMARCAL1 **(A)**. Co-localization of GFP- ΔHSA with endogenous SMARCAL1 in the absence of doxorubicin treatment **(B)** Co-localization of GFP- ΔHSA with endogenous SMARCAL1 in the presence of doxorubicin treatment **(C)**. Pearson’s coefficient for the interaction of GFP-ΔHSA with endogenous SMARCAL1 in the absence of doxorubicin treatment **(D)**. Pearson’s coefficient for the interaction of GFP-ΔHSA with endogenous SMARCAL1 in the presence of doxorubicin treatment **(E)**. Co-localization of GFP-ΔN and GFP-ΔC with endogenous SMARCAL1 was monitored in the absence of doxorubicin treatment **(F)**. Co-localization of GFP-ΔN and GFP-ΔC with endogenous SMARCAL1 was monitored in the presence of doxorubicin treatment **(G)**. Pearson’s coefficient for the interaction of GFP-ΔN and GFP-ΔC with endogenous with SMARCAL1 in the absence of DNA damage **(H)**. Pearson’s coefficient for the interaction of GFP-ΔN and GFP-ΔC with endogenous with SMARCAL1 in the presence of DNA damage. In all these experiments, HeLa cells were treated with 2 μM doxorubicin for 10 min and n ≥ 40 cells were analyzed. Star indicates significance with *p-value < 0.05, **p-value < 0.005, ***p value <0.0001. The wild-type data used in the analysis has been shown inFigure 2.The scale in the images is 20 μm.

**Multiple regions of BRG1 are required for interaction with SMARCAL1:** To study the interaction of BRG1 with SMARCAL1, three deletion constructs of BRG1 were made- ΔHSA lacking the HSA domain, ΔN lacking the entire N-terminus domain, and ΔC lacking the C-terminal domain containing the helicase motifs (Supplementary Figure 5B). Co-localization experiments showed that none of the deletion mutants of BRG1 were able to interact with SMARCAL1 both in the absence and presence of DNA damage (Figure 5A–H). Further, the Pearson’s correlation values of BRG1 mutants were either equal to the vector-only control or showed a negative correlation (Figure 5A–H).

Thus, the HARP domains of SMARCAL1 are required for interaction with BRG1 while multiple regions of BRG1 possibly mediate the interaction with SMARCAL1.

**Mutations associated with SIOD and CSS4 impairs the co-localization:** Mutations in SMARCAL1 cause Schmike Immuno‐osseous Dysplasia (SIOD) while mutations in BRG1 are associated with Coffin-Siris Syndrome (CSS4) (Boerkoel et al., 2002; Tsurusaki et al., 2012).

**FIGURE 6:**
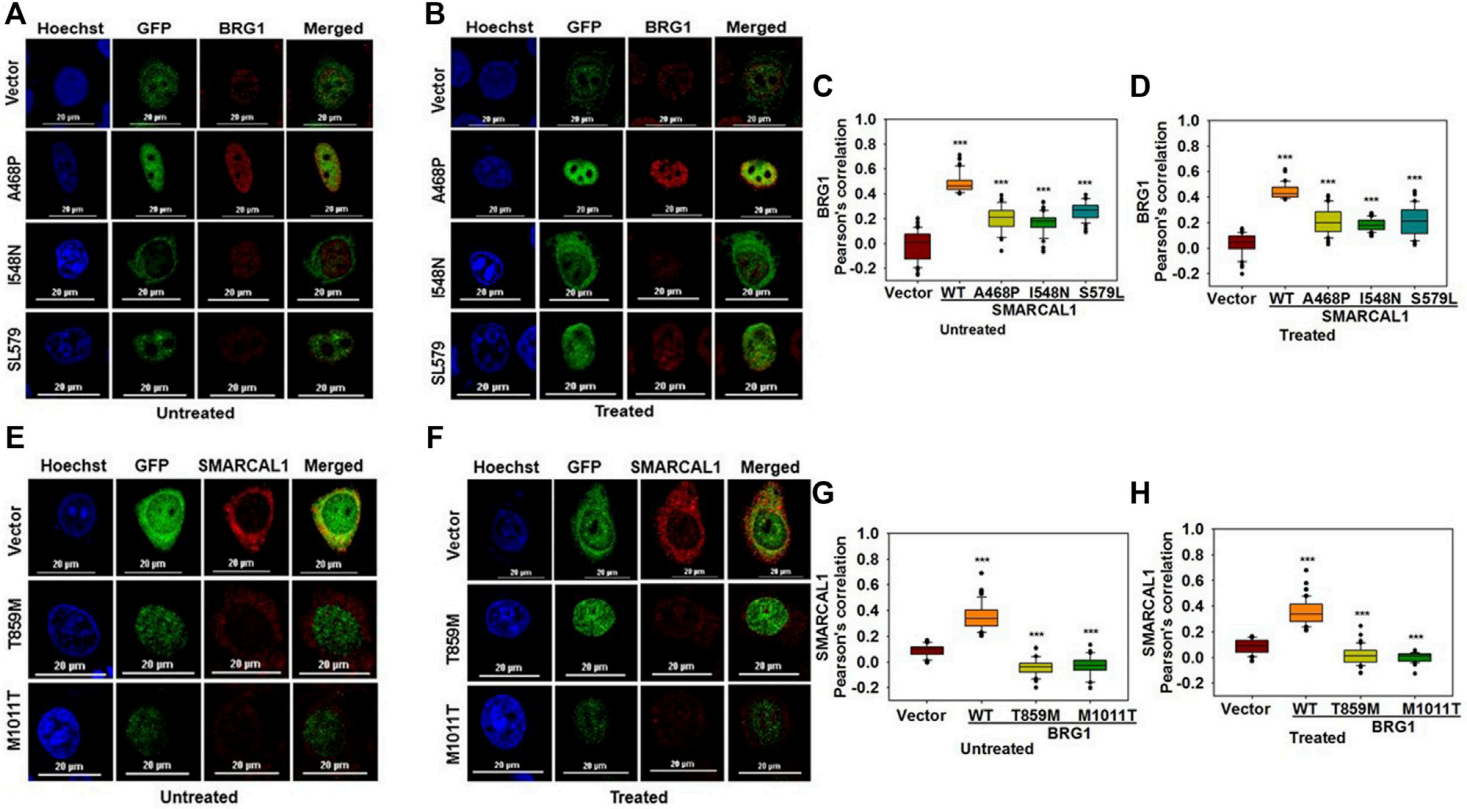
Mutations associated with SIOD and CSS4 impairs the co-localization **(A)**. Co-localization of GFP-SMARCAL1-A468P, GFP-SMARCAL1-I548N, and GFP-SMARCAL1-S579L with BRG1 in the absence of doxorubicin treatment **(B)**. Co-localization of GFP-SMARCAL1-A468P, GFP-SMARCAL1-I548N, and GFP-SMARCAL1-S579L with BRG1 in the presence of 2 μM doxorubicin treatment for 10 min **(C)**. Pearson’s coefficient for the interaction in the absence of doxorubicin treatment **(D)**. Pearson’s coefficient for the interaction in the presence of doxorubicin treatment **(E)**. Co-localization of GFP-BRG1-T859M and GFP-BRG1-M1011T with SMARCAL1 in the absence of doxorubicin treatment **(F)**. Co-localization of GFP-BRG1-T859M and GFP-BRG1-M1011T with SMARCAL1 in the presence of doxorubicin treatment **(G)**. Pearson’s coefficient for the interaction in the absence of doxorubicin treatment **(H)** Pearson’s coefficient for the interaction in the presence of doxorubicin treatment. In all these experiments, HeLa cells were treated with 2 μM doxorubicin for 10 min and n ≥ 60 cells for GFP-SMARCAL1-A468P and BRG1, n ≥ 50 cells for GFP-SMARCAL1- I548N and BRG1, and n ≥ 90 cells for GFP-SMARCAL1-S579L and BRG1 were analyzed. Star indicates significance with *p-value < 0.05, **p-value < 0.005, ***p value <0.0001. The wild-type data used in the analysis has been shown in Figure 2. The scale in the images is 20 μm.

To understand whether mutations that cause SIOD also lead to loss of co-localization, we studied the interaction of three mutations present in SIOD patients-A468P, I548N, and S579L with BRG1. All these three mutants are present in the Rec A-like Domain1 and cannot hydrolyze ATP (Gupta et al., 2015). HeLa cells were transfected with constructs expressing these three mutant proteins and the co-localization with BRG1 was analyzed in the absence and presence of DNA damage. Experimental results showed that the co-localization between the mutant SMARCAL1 proteins and BRG1 decreased as compared to the wild-type SMARCAL1 and BRG1 both in the absence and presence of DNA damage (Figure 6A–D), suggesting that the mutations have impaired the interaction between the two proteins.

Next, to understand whether CSS4-associated mutants can interact with SMARCAL1, the co-localization of two CSS4-associated mutants-T859M and M1011T-with SMARCAL1 was studied. Experimental results showed that neither of the two mutant proteins was able to co-localize with SMARCAL1 or showed a negative correlation (Figure 6E–H).

Thus, mutations that cause SIOD4-or CSS4- lead to reduced co-localization, indicating that phenotypes observed in these syndromes might also be a consequence of the loss in protein-protein interaction.

## Discussion

The DNA damage response pathway begins with the sensing of the DNA damage, followed by the recruitment of proteins to the site of DNA damage. The ATP-dependent chromatin remodeling proteins are recruited to the site of DNA damage wherein they remodel the chromatin allowing for the repair process to occur. For example, RSC, an ATP-dependent chromatin remodeler in *S. cerevisiae*, has been found to be recruited to the DSB generated by HO endonuclease at the *MAT* locus wherein it mediates H2A phosphorylation as well as strand resection (Kent et al., 2007). INO80, another ATP-dependent chromatin remodeler, too has been shown to be recruited to DSB generated by HO endonuclease in *S. cerevisiae* (Tsukuda et al., 2005; Panday et al., 2015).

BRG1 and SMARCAL1, both members of the ATP-dependent chromatin remodeling protein family, are known to participate in the repair process. Both have been shown to co-localize with γH2AX, considered as one of the markers of DNA damage (Rogakou et al., 1998; Fillingham et al., 2006). Studies have shown that SMARCAL1 interacts with RPA (Ciccia et al., 2009) and mediates fork regression (Bétous et al., 2012) while BRG1 has been shown to modulate DNA double-strand break repair (Park et al., 2006; Lee et al., 2010; Qi et al., 2015). Studies have also shown that in HeLa cells, SMARCAL1 and BRG1 transcriptionally co-regulate each other on induction of doxorubicin-mediated DNA damage (Haokip et al., 2016). This transcriptional co-regulation is required for the recruitment of 53BP1 and thus, for DNA damage repair (Patne et al., 2017; Sethy et al., 2018).

In this paper, we have now shown that SMARCAL1 and BRG1 interact with each other directly both in the absence and presence of DNA damage. The HARP domains of SMARCAL1, which are known to mediate the annealing helicase activity of the protein (Ghosal et al., 2011), are needed for interaction with BRG1 and thus, suggesting that these domains might have an additional function in mediating the protein-protein interaction. In contrast, a single domain of BRG1 could not be identified. The experimental results demonstrate that multiple regions of the protein might be involved in the interaction with SMARCAL1.

The defining feature of the ATP-dependent chromatin remodeling proteins is the ATPase activity they exhibit in the presence of DNA/nucleosome substrate (Quinn et al., 1996; Muthuswami et al., 2000). The ATPase activity, we show, is also required for the protein-protein interaction both in the absence and presence of doxorubicin-induced DNA damage. Thus, the ATPase dead mutant of SMARCAL1, K464A (Gupta et al., 2015), fails to interact with BRG1. Similarly, the ATPase dead mutant of BRG1, K785R (Khavari et al., 1993), showed impaired interaction with SMARCAL1. This was intriguing and led us to examine the interaction in SIOD-and CSS4-associated mutants. Co-localization studies showed that the interaction of the SIOD-associated mutants with BRG1 was impaired. Similarly, the CSS4-associated mutants showed impaired co-localization with SMARCAL1. The SIOD-associated mutants lie outside the HARP domain. Studies using ADAAD, the bovine homolog of SMARCAL1, have shown that these residues are needed for maintaining the global conformation of the protein (Gupta et al., 2015). Thus, the loss in the interaction with the cognate protein partner might be a consequence of the altered conformation of the mutant proteins.

The interaction of BRG1 and SMARCAL1 is interesting because both are ATP-dependent chromatin remodeling proteins. Though two ATP-dependent chromatin remodeling proteins have been shown to mediate gene regulation of the same subset of genes (Patne et al., 2017; Yang et al., 2017; Sethy et al., 2018), and proteomic studies have identified that two ATP-dependent chromatin remodeling proteins might be interacting (Rowbotham et al., 2011), this is the first study validating the interaction between two ATP-dependent chromatin remodeling proteins. We hypothesize that the interaction between the two proteins is required for transcriptional co-regulation of genes both in the absence and presence of DNA damage. The loss of interaction observed in both SIOD-associated and CSS4-associated mutants might be one of the reasons for the observed pathophysiology of these diseases.

The importance of the ATPase activity for this interaction is interesting but not surprising. Previously it has been shown that the ATPase activity of both BRG1 and SMARCAL1 is needed for the transcriptional regulation of *ATM*, *ATR*, *DROSHA*, *DGCR8* and *DICER* on induction of DNA damage by doxorubicin treatment in HeLa cells. ChIP-reChIP experiments showed that both BRG1 and SMARCAL1 are present simultaneously on the promoter (Haokip et al., 2016; Patne et al., 2017; Sethy et al., 2018). Further, mutations in the helicase motifs in SMARCAL1 have been shown to cause alterations in the protein conformation (Nongkhlaw et al., 2012; Gupta et al., 2015; Bansal et al., 2018). Thus, it is possible that the ATPase dead mutants of BRG1 and SMARCAL1 have altered protein conformation that precludes the interaction between them.

In the presence of DNA damage, BRG1 and SMARCAL1, possibly together, with γH2AX, mediate DNA damage response. Though now we know that SMARCAL1-γH2AX, BRG1-γH2AX, and SMARCAL1-BRG1 co-localize, this experimental setup did not allow us to show whether the proteins are present simultaneously at the site of DNA damage. Therefore, we can only hypothesize that the three proteins are possibly forming a trimeric complex at the site of DNA damage. The direct interaction between SMARCAL1-γH2AX or BRG1-γH2AX to form a trimeric complex needs to be confirmed in future. The other avenue for exploration is to delineate whether BRG1 in complex with SMARCAL1 and/or γH2AX is post-translationally modified. In our experiments, we found that SMARCAL1 pulls down BRG1 that is of higher molecular weight while this band is absent when the protein is immunoprecipitated with antibodies against γH2AX. BRG1 is known to be modified by ATM (Kwon et al., 2015). Further, pATM has been shown to co-localize with BRG1 on *ATM, ATR, DROSHA, DGCR8* and *DICER* promoters (Sethy et al., 2018). High-resolution mass spectrometry has also identified that BRG1 can be acetylated; however, the relevance of acetylation has not yet been understood (Choudhary et al., 2009). We, therefore, hypothesize that in the cells there could be at least two forms of BRG1 complex. In one complex, BRG1 is possibly post-translationally modified while in the other complex it is in unmodified form. For example, it is possible that on the promoters, where BRG1 is in the same space with SMARCAL1 and pATM, it is phosphorylated by ATM. Further experiments are needed to decipher the modification and the relevance with respect to function.

It has been recently shown that loss-of-function mutations in *Fancm* and *Brca1* leads to synthetic lethality (Panday et al., 2021). Like SMARCAL1, FANCM and BRCA1 are also required for repair of stalled replication fork. Studies have shown that depletion of SMARCAL1 in BRCA1/2 deficient cells leads to reduction in genomic instability (Taglialatela et al., 2017). It is, thus, possible that a similar synthetic lethality exists between SMARCAL1 and BRG1, that can be exploited for generation of small molecule inhibitors for cancer. Indeed, one such molecule, Active DNA-dependent ATPase A inhibitor (ADAADi) targets the ATPase domain of both SMARCAL1 and BRG1 and has been shown to be effective against breast cancer cells lines as well as prostate tumors developed in mouse models (Dutta et al., 2012; Wu et al., 2016; Muthuswami et al., 2019). Identification of many more such molecules might help in augmenting the repertoire of inhibitors leading to development of chemotherapeutic drugs.

## Data Availability

The datasets presented in this study can be found in online repositories. The names of the repository/repositories and accession number(s) can be found below: https://www.ncbi.nlm.nih.gov/geo/, GSE137250.
