# Supplementary figures and images for: On the Interaction Between SMARCAL1 and BRG1
Source: Front Cell Dev Biol. 2022 Jun 16;10:870815. doi: 10.3389/fcell.2022.870815 (PMC9243424; doi:10.3389/fcell.2022.870815)

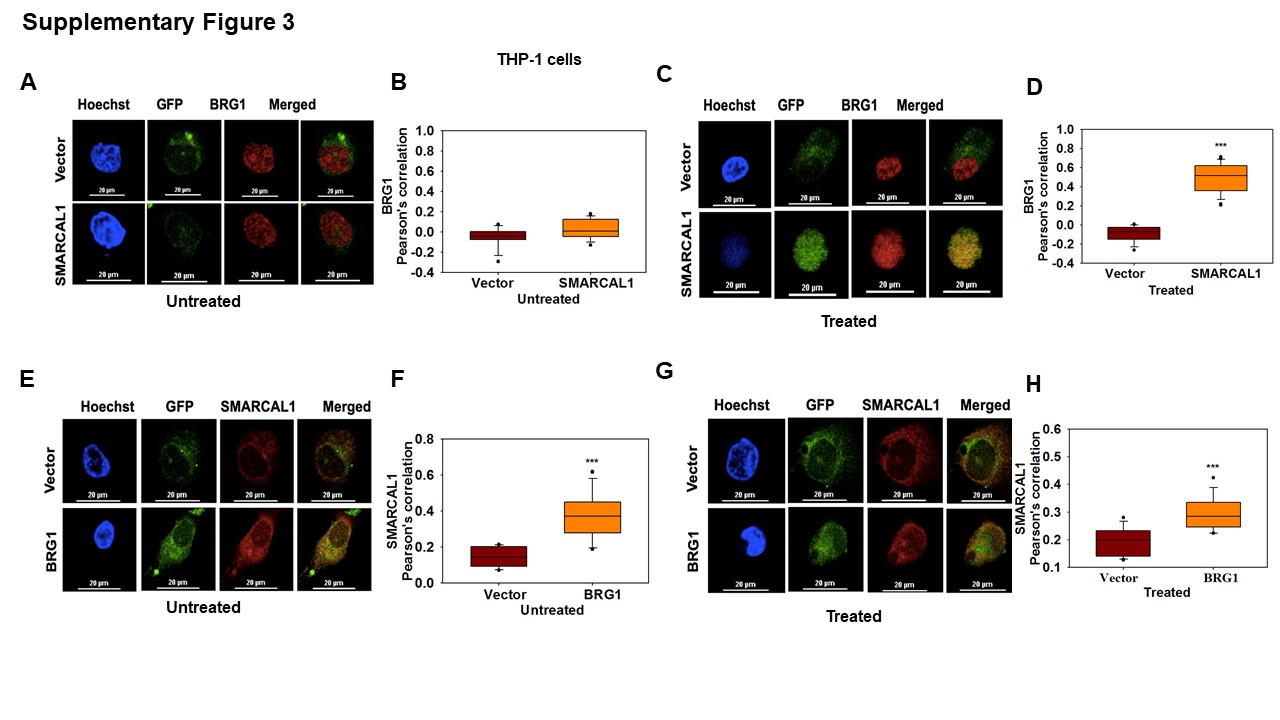

Supplement: Supplementary file 1 [file Image3.JPEG]

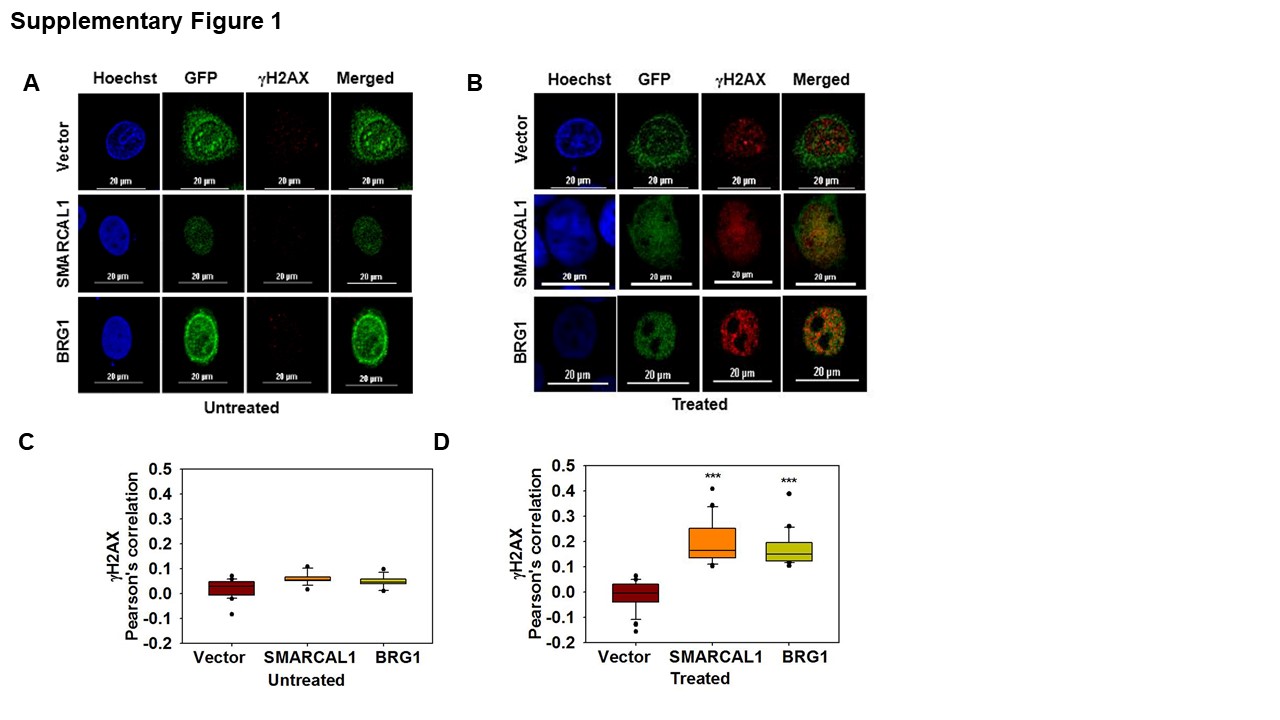

Supplement: Supplementary file 3 [file Image1.JPEG]

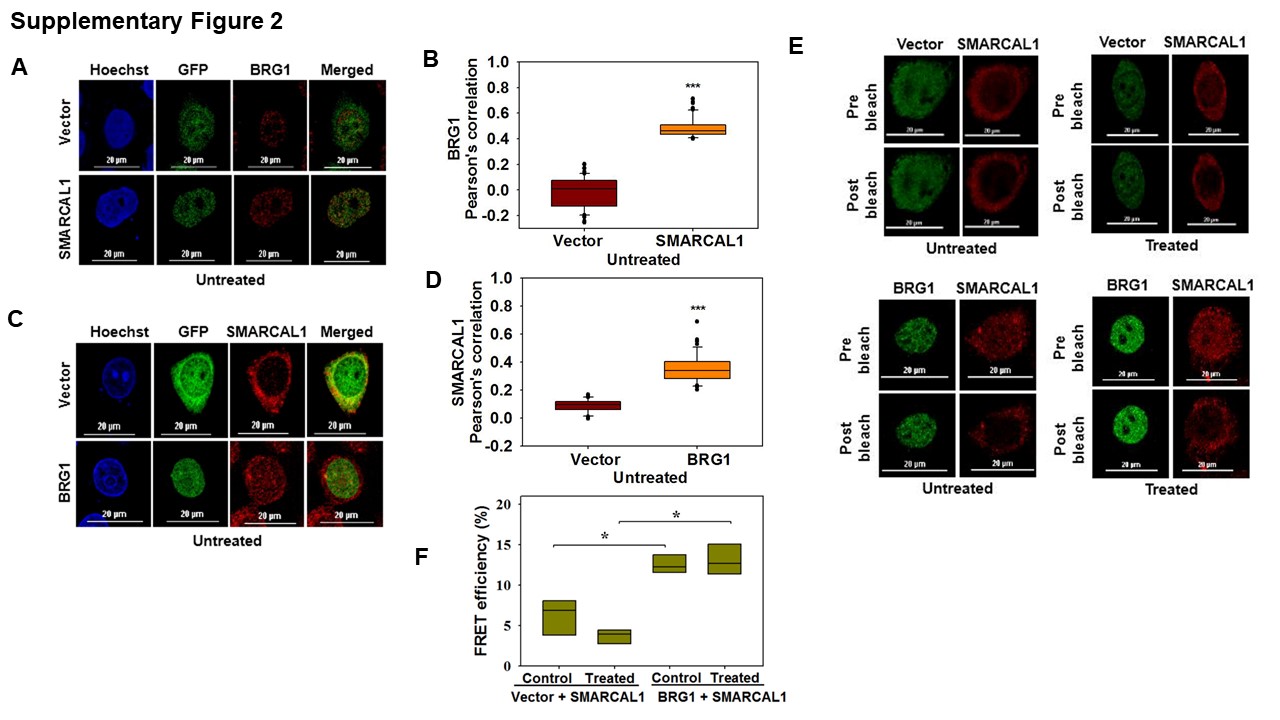

Supplement: Supplementary file 4 [file Image2.JPEG]

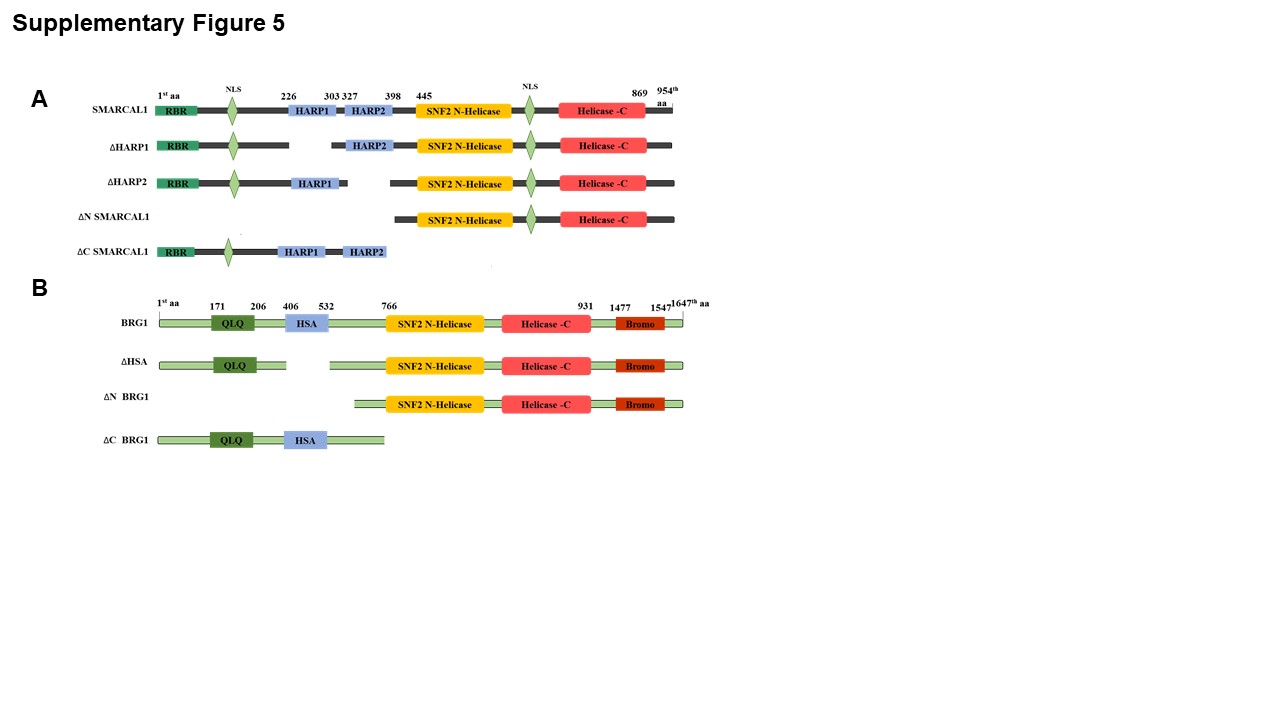

Supplement: Supplementary file 5 [file Image5.JPEG]

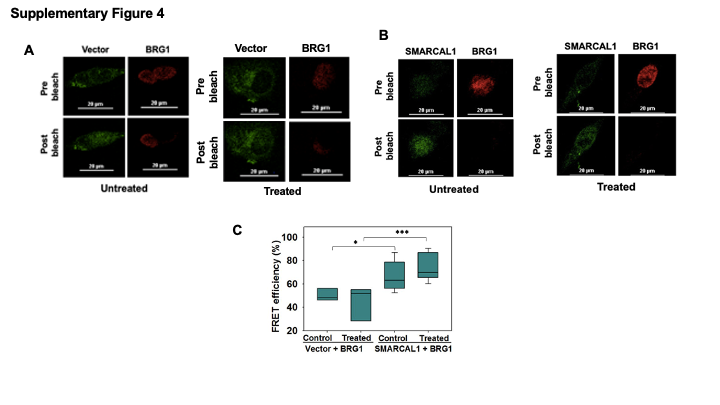

Supplement: Supplementary file 6 [file Image4.tiff]
