# Supplementary material for: On the Interaction Between SMARCAL1 and BRG1
Source: Front Cell Dev Biol. 2022 Jun 16;10:870815. doi: 10.3389/fcell.2022.870815 (PMC9243424; doi:10.3389/fcell.2022.870815)
Supplement: Supplementary file 2 [file Table1.DOCX]

**Supplementary Table 1: Primers (5' to 3') used in cloning deletion constructs and mutants.**

| **Mutant** | **Forward Primer (5' to 3')** | **Reverse Primer (5' to 3')** |
| --- | --- | --- |
| **SMARCAL1 deletion constructs** | | |
| **ΔHARP1** | AGCTGGGCCTATGGCAGCAGCGAGTCACCC | AGCCTTCTGCTGGAGTCTTCCTTCTGTCCT |
| **ΔHARP2** | AGCACGACTCTCACCCTGGCGTTTGCTTCT | AGCTGGAGCTGATGGAAGGCCGGCCTGTCC |
| **ΔC** | AACTCGAGATG TCCTTGCCTCTTACAGAGGAGC | TGGGCCCTACATCTGGCGTGAGACTGAGAGATG |
| **ΔN** | AACTCGAGCCAGAGGCAGACCTTTCTGAAGTGG | TGGGCCCTTACAGGGGAGACGTAAAGCTGTCCC |
| **BRG1 deletion constructs** | | |
| **ΔHSA** | AGCGAAGATGAGGAGGGGTACCGCAAGCTC | AGCCTGCTTCTCCAGCTTCTCAGTGATGCG |

**SUPPLEMENTARY FIGURE LEGENDS**

**Supplementary Figure 1. SMARCAL1-γH2AX and BRG1-γH2AX interact in the presence of doxorubicin-induced DNA damage.** Co-localization between GFP-SMARCAL1-γH2AX and GFP-BRG1-γH2AX was monitored (A) in the absence and (B) in the presence of doxorubicin-induced DNA damage in HeLa cells. (C). Pearson’s coefficient for SMARCAL1-γH2AX and BRG1-γH2AX plotted with vector only control.

In all these experiments, HeLa cells were treated with 2 μM doxorubicin for 10 minutes and n≥15 cells were analyzed. Star indicates significance with *p value < 0.05, **p value < 0.005, ***p value < 0.0001. The scale in the images is 20 μm.

**Supplementary Figure 2. SMARCAL1 and BRG1 interact with each other both in the absence and presence of doxorubicin-induced DNA damage:** (A). Co-localization between GFP-SMARCAL1 and endogenous BRG1 was monitored in the absence of doxorubicin-induced DNA damage in HeLa cells. (B). Pearson’s coefficient for SMARCAL1-BRG1 plotted with vector only control. (C). Co-localization between GFP-BRG1 and endogenous SMARCAL1 was monitored in the absence of doxorubicin-induced DNA damage in HeLa cells. (D). Pearson’s coefficient for BRG1-SMARCAL1 plotted with vector only control. Acceptor Photobleach FRET efficiency showing the interaction between BRG1 and SMARCAL1. (E). FRET showing no change in GFP-vector signal after bleaching endogenous BRG1 in control HeLa cell, and in doxorubicin treated cell. FRET showing the increase in GFP-BRG1 signal after bleaching endogenous SMARCAL1 in control HeLa cell, and in doxorubicin treated cell. (F). FRET efficiency showing the interaction between GFP-BRG1 and SMARCAL1.

In the co-localization experiments, HeLa cells were treated with 2 μM doxorubicin for 10 minutes and n≥90 cells for GFP-SMARCAL1 and BRG1, and n≥40 cells for GFP-BRG1 and SMARCAL1 were analyzed. In the FRET experiments, n≥8 cells were analyzed. Star indicates significance with *p value < 0.05, **p value < 0.005, ***p value < 0.0001. The scale in the images is 20 μm.

**Supplementary Figure 3. SMARCAL1 and BRG1 co-localize with each other in THP-1 cells:** (A). Co-localization between GFP-BRG1 and endogenous SMARCAL1 was monitored in the absence of doxorubicin-induced DNA damage in THP-1 cells. (B). Pearson’s coefficient for BRG1-SMARCAL1 plotted with vector only control. (C). Co-localization between GFP-BRG1 and endogenous BRG1 was monitored in the presence of doxorubicin-induced DNA damage in HeLa cells. (D). Pearson’s coefficient for BRG1-SMARCAL1 plotted with vector only control. (E). Co-localization between GFP-SMARCAL1 and endogenous BRG1 was monitored in the absence of doxorubicin-induced DNA damage in HeLa cells. (F). Pearson’s coefficient for SMARCAL1-BRG1 plotted with vector only control. (G). Co-localization between GFP-SMARCAL1 and endogenous BRG1 was monitored in the presence of doxorubicin-induced DNA damage in HeLa cells. (H). Pearson’s coefficient for SMARCAL1-BRG1 plotted with vector only control. In the co-localization experiments, THP-1 cells were treated with 2 μM doxorubicin for 10 minutes and n≥15 cells were analyzed. Star indicates significance with *p value < 0.05, **p value < 0.005, ***p value < 0.0001. The scale in the images is 20 μm.

**Supplementary Figure 4: SMARCAL1 and BRG1 interact with each other in THP-1 cells:**

Acceptor Photobleach FRET efficiency showing the interaction between BRG1 and SMARCAL1. (A). FRET performed in vector control cells in untreated and in doxorubicin treated THP-1 cells. (B). FRET showing increase in the GFP-SMARCAL1signal after bleaching endogenous BRG1 in untreated and in doxorubicin treated THP-1 cells. (C). FRET efficiency showing the interaction between GFP-SMARCAL1 and BRG1.

In the FRET experiments, n≥9 cells were analyzed. The scale in the images is 20 μm.

**Supplementary Figure 5. Different domains of SMARCAL1 and BRG1 are required for mediating interaction with each other:** (A) Schematic diagram domains present in SMARCAL1 and its mutants. (B) Schematic diagram domains present in BRG1 and its mutants.
